# The ‘Yin and Yang’ of Cancer Cell Growth and Mechanosensing

**DOI:** 10.3390/cancers13194754

**Published:** 2021-09-23

**Authors:** Malak Amer, Lidan Shi, Haguy Wolfenson

**Affiliations:** Department of Genetics and Developmental Biology, Rappaport Faculty of Medicine, Technion—Israel Institute of Technology, Haifa 31096, Israel; malaka@campus.technion.ac.il (M.A.); lidan.shi@campus.technion.ac.il (L.S.)

**Keywords:** mechanosensing, anchorage-independence, rigidity sensing, tumor stiffness, ECM

## Abstract

**Simple Summary:**

Cells can sense their physical environment, or extracellular matrix (ECM), in a process called ‘mechanosensing’. They do this via adhesion sites which allow them to attach to the ECM and to simultaneously obtain information about its mechanical properties. This mechanical information is converted to biochemical signals that affect cell behavior in various ways. Stiff surroundings tend to promote proliferation of normal cells, whereas soft substrates can lead to their death. Cancer cells, however, can survive on soft substrates, while maintaining increased proliferation on stiff surfaces. This observation is interesting because it suggests that cancer cells potentially ignore negative physical cues, such as a soft ECM, and exploit positive stimuli in the form of a stiff ECM. This brings to mind a ‘yin and yang’ equilibrium which is ever shifting in favor of cancer cell survival and growth. We propose that different rigidities, which cancer cells may encounter in different regions of the body, can lead to modifications in the interactions and adhesions of the cells with the ECM, thus activating signaling pathways that boost cancer cell proliferation. Here, we discuss these interactions through the lens of mechanosensing, and its abnormal function in cancer.

**Abstract:**

In cancer, two unique and seemingly contradictory behaviors are evident: on the one hand, tumors are typically stiffer than the tissues in which they grow, and this high stiffness promotes their malignant progression; on the other hand, cancer cells are anchorage-independent—namely, they can survive and grow in soft environments that do not support cell attachment. How can these two features be consolidated? Recent findings on the mechanisms by which cells test the mechanical properties of their environment provide insight into the role of aberrant mechanosensing in cancer progression. In this review article, we focus on the role of high stiffness on cancer progression, with particular emphasis on tumor growth; we discuss the mechanisms of mechanosensing and mechanotransduction, and their dysregulation in cancerous cells; and we propose that a ‘yin and yang’ type phenomenon exists in the mechanobiology of cancer, whereby a switch in the type of interaction with the extracellular matrix dictates the outcome of the cancer cells.

## 1. Introduction

A decade ago, Bissell and Hines put forward the question “Why don’t we get more cancer?”, and proposed that the tumor microenvironment can either restrain or promote cancer progression, depending on its context [1]. Since then, numerous studies have emphasized the roles of the stroma in cancer progression, and a central subset of these focused on the mechanobiological processes that are involved. Primarily, high tumor stiffness, which results from high stromal extracellular matrix (ECM) stiffness, was shown to be common in different cancer types, including breast [2], pancreatic [3], colorectal [4] and liver cancer [5], among others. This led to reinforcement of the theory that high stiffness promotes cancer progression, with evidence showing that it enhances tumor growth, epithelial-to-mesenchymal transition (EMT) and escape of metastatic cells from the primary tumor site [6,7]. On the other hand, cancer cells can also thrive in very soft environments, a feature that is termed ‘anchorage-independence’, and is typically validated using soft agar assays, in conditions that do not support strong adhesion. Also, recent findings suggest that there may in fact be contradicting roles for the ECM stiffness, as in some cases attempting to target the ECM as a therapeutic strategy can lead to detrimental outcomes [8].

Thus, it has become abundantly clear that the stroma, and in particular the tumor ECM, provides substantial signals, either inhibitory or excitatory, that affect cancer progression. Here we discuss the processes of mechanosensing and mechanotransduction, and describe how their dysregulation in cancer affects cancer progression. We conclude by proposing that the dual ability of cells to exploit high stiffness in the primary tumor site while also being able to survive and grow under anchorage-independent conditions may stem from an alteration in their adhesive properties.

## 2. The ECM Provides the Biomechanical Context of Tissues and Cells

Different tissues and organs have distinct biomechanical properties which are altered throughout development, wound healing or disease [9]. Consequently, all cell types are functionally attuned to the mechanical properties of their tissues of origin. The physical contact of adherent cells with their environment supports their normal growth, survival, differentiation and morphogenesis, and when deprived of this contact, cells undergo anoikis, an anchorage-dependent form of apoptosis [10]. In the event of cellular detachment from the tissue of origin, anoikis prevents the attachment and eventual colonization of an unsuitable matrix. The functional dependence of cells on the mechanical properties of the environment is also manifested in multicellular organization of cells. For example, mammary epithelial cells that interact with a relatively soft matrix in vivo, spontaneously assemble acini similar to those found in normal breast tissue when grown ex vivo on a comparably soft substrate; importantly, they lose this ability on stiffer matrices [6]. Thus, the mechanical properties of the ECM play a considerable role in determining cell fate, a process which is driven in large via internal cytoskeletal changes that result in altered cellular tension. This in turn, can affect the mechanical properties of the ECM, highlighting the crosstalk between cells and their surroundings [11].

The ECM is comprised of a multitude of proteins, polysaccharides, and their derivatives. Their respective proportions, post-translational modifications, degree of crosslinking and arrangements dictate tissue properties [12]. Typically, ECM proteins are synthesized and secreted into the interstitial matrix by fibroblasts that reside within the tissue matrix [13]. ECM proteins include structural proteins, such as collagens and fibronectin, which contribute to the mechanical form of the ECM, and matricellular proteins, such as osteonectin (SPARC), which regulate signaling pathways [14]. In addition to providing structural support and serving as a protective layer from external stresses, the ECM network also has the capacity to capture small molecules, such as growth factors or cytokines, either by directly binding them or by confining them. Upon matrix degradation, the sequestered factors are released, leading to an increase in their bioavailability to nearby cells [15]. Furthermore, ECM proteins may act as ligands for cell surface receptors, in particular integrin molecules, which upon binding and clustering, trigger signal transduction pathways (see more below). Therefore, the cellular expression of specific receptors, combined with the presence of complementary ECM proteins, affects cell signaling and behavior, and ultimately tissue function [16].

## 3. The Mechanical Tumor Microenvironment Affects Tumor Growth

The ECM architecture is regularly restructured through a continuous and dynamic cycle of disintegration and reformation known as ECM remodeling. This process is an integral part of tissue homeostasis and function, and is modulated throughout development and healing processes. The delicate interplay between matrix synthesis, degradation and modification is regulated by enzymes such as proteases, matrix metalloproteases (MMPs) and their inhibitors [17,18]. Chronic impairment of ECM remodeling is characteristic of cancer, and is associated with a pathologically altered biochemical and biomechanical matrix [19,20]. For example, in normal breast tissue, MMPs are tightly controlled in ECM remodeling for mammary gland growth and involution [12]. In cancer, however, this control is lost, and some MMPs are overexpressed in the tumor stroma or in transformed mammary epithelial cells. Importantly, this aberrant activity was suggested to play a causative role in malignancy, both by enhancing ECM branching and desmoplasia, and by allowing tumor cell dissemination [21]. Lysyl oxidase (LOX), an enzyme which cross-links newly synthesized collagen molecules, is upregulated in response to elevated collagen deposition, and its overexpression has been shown to correlate with metastasis and decreased survival in cancer patients [22,23]. Another cancer-associated ECM protein is tenascin C (TNC), a matricellular hexameric glycoprotein that binds to ECM proteins, such as fibronectin, and their cell membrane receptors, thereby altering the affinity between the two [24]. TNC was found to be highly expressed by breast cancer cells, and to promote metastases formation in the lung [25]. Furthermore, fibrotic stromal matrix proteins (such as collagen type I, III and V, elastin, vitronectin, matricellular proteins and oncofetal fibronectin) are overexpressed in breast cancer. This is accompanied by the upregulation of glycosaminoglycans such as hyaluronan and chondroitin sulfate, and downregulation of collagen type IV and LM-111. The above examples are just a few of many that highlight the extensive remodeling of the ECM in cancer. For detailed reviews on ECM composition and remodeling in cancer, see Cox and Erler 2011, Oskarsson 2013 [16,26].

The finding of a clear correlation between breast tissue density and an increased risk of developing breast cancer drew attention to the relationship between tissue rigidity and cancer [27]. Detailed studies of the spatial distribution of stiffnesses of human breast tissues showed that normal and benign tissues had consistent profiles characterized by one narrow rigidity range (peak), whereas malignant tissues displayed a broad stiffness distribution [2]. Increased crosslinking, as well as particular arrangements of the ECM in tumor tissues, were found to cause this stiffness heterogeneity in the tumor microenvironment, with the highest stiffness being at the invasive front [28]. Numerous stages of cancer progression have been implicated in the attempts to explain the link between high tissue stiffness and poor patient prognosis. For example, high stiffness was shown to enhance EMT [6,7], hyper-activate signaling pathways [29], promote cancer cell-endothelium interactions [30], drive invasion [31], enhance cell migration (durotaxis) [32,33], and prevent infiltration of immune cells into the tumor microenvironment [34]. Nevertheless, the most prominent effect of matrix stiffness appears to be on cell proliferation, which is the basic cellular function that is disrupted in cancer, and which initiates cancer formation. In one of the early studies that addressed the link between matrix rigidity and cancer cell growth, a comparison between the rigidity-dependent growth of normal and H-ras transformed fibroblasts (NIH-3T3) was performed. In this study, normal cells exhibited decreased proliferation and increased apoptosis rates on soft collagen-coated polyacrylamide substrates compared to stiff, while their transformed counterparts maintained the same growth patterns regardless of substrate rigidity [35]. The ability to (at least) avoid apoptosis on soft matrices, while growing rapidly on stiff ones was observed in numerous cancer types. For example, in hepatocellular carcinoma cells (HCCs), higher substrate stiffness was found to promote proliferation and chemotherapy resistance through β1-integrin and FAK, while lower adhesive conditions promoted their dormancy [36]. Glioma cells cultured on fibronectin-coated polymeric ECM substrates proliferated more rapidly on rigid surfaces compared to compliant surfaces [37], and lung adenocarcinoma cells had increased proliferation when the ECM stiffness was increased, in a physiological range and independently of ECM composition [38]. Increasing ECM stiffness by enhancing collagen crosslinking via LOX modulation also increased breast tumor survival and proliferation [29]. Interestingly, normalizing the tensional homeostasis of tumor cells could revert them towards a non-malignant phenotype, demonstrating the functional link between matrix mechanical properties, and normal cell behavior [39].

Thus, a common theme appeared to emerge in which cancer cells can exploit high stiffness for growth, while avoiding apoptosis or even being able to grow on low stiffnesses. However, in recent clinical studies, “softening” the stiff ECM in pancreatic ductal adenocarcinoma (PDAC) proved to be detrimental, contrary to expectations [8], and several studies showed that stromal fibroblasts (which secrete the matrix) can either promote or restrain cancer progression [40]. Moreover, desmoplasia reduction in PDAC mouse models (via deletion of Sonic hedgehog from stromal fibroblasts), led to increased metastasis [41]. These findings do not rule out the growth-related mechanical effects, but suggest that in some cases, a decrease in stiffness may in fact promote certain aspects of cancer progression.

The promotion of growth is often closely linked to the activation of Yes-associated protein (YAP) and TAZ, which are transcriptional coactivators and core components of the Hippo pathway. In addition to having roles in tissue development and homeostasis, YAP/TAZ act as mechanosensors of the ECM [42]. They are activated by increased stiffness to promote the production of profibrotic mediators and ECM proteins, which leads to an additional increase in tissue stiffness. This in turn activates YAP/TAZ further, thus creating a feed-forward loop which can result in tissue fibrosis. Indeed, aberrations in YAP/TAZ activity have been found both in cancer and fibrosis in humans and in animal models [43]. In epithelial cells, YAP/TAZ activation by increased ECM stiffness promotes their proliferation and survival, and the involvement of YAP/TAZ in EMT also contributes to tumor progression [44].

## 4. ECM Mechanosensing Is a Multi-Step Process

In further investigations of the link between rigidity and cancer cell growth, a screen of numerous cancer cell lines showed that they can be categorized as rigidity-dependent (e.g., MDA-MB-231) or independent (e.g., mPanc96) for proliferation [45]. With increasing matrix rigidity, most of the tested cells were rigidity-dependent and had higher proliferation rates, while rigidity-independent cells had consistent proliferation rates across the range of rigidities tested. The existence of the latter population might seem to contradict the view that stiffness promotes proliferation; however, more intriguing is the loss of growth inhibition on soft matrices in these cells [45]. In particular, their growth even under anchorage-independent conditions indicates that the ability of such cells to properly sense ECM rigidity is impaired.

Sensing matrix mechanical signals occurs through integrin adhesions, which are the major sites of contact between cells and the ECM. At the heart of these structures are integrin complexes that mediate the connection [46]. Integrins are transmembranal heterodimeric receptors containing α and β subunits, of which there are 18 and 8 identified subtypes, respectively. Different combinations of the various integrin subtypes enable the recognition and binding of specific ECM proteins, such as fibronectin, collagens and laminins [47]. When integrins bind to ECM proteins, intracellular structural and signaling proteins are recruited to the adhesion. The signaling proteins include kinases such as Src, focal adhesion kinase (FAK) and integrin-linked kinase (ILK), as well as phosphatases such as receptor-like tyrosine phosphatase α (RPTP-α) [48]. A multitude of proteins, such as talin, vinculin, paxillin and zyxin, bridge the integrin receptors to the actin cytoskeleton [49]. Thus, a physical link is forged between the ECM and the cytoskeleton, making integrin adhesions pivotal to cellular mechanosensing of signals from inside and outside the cell [50,51].

Even though the mechanisms of mechanosensing are still being explored, there is a growing understanding that the process occurs on multiple temporal and spatial levels. The common thread among these is the link between ECM rigidity, cytoskeletal forces, and adhesion size/stability. Mechanosensing is initiated following early kindlin- and talin-mediated activation and clustering of integrin dimers [52], which leads to the formation of nascent adhesions, typically in lamellipodial regions of the cell. This stage is considered to be force-independent, as such clusters can form in cells on supported lipid bilayers that do not provide resistance to cell-generated forces [53], as well as in cells treated with blebbistatin which inhibits actomyosin contractility [54]. The linkage of the integrin cytoplasmic tails to actin through talin is a prerequisite for the next stage, which is force-dependent. At this stage, myosin-driven local contractions of the matrix via neighboring adhesion sites determine whether the cell will reinforce the adhesions, an outcome which is contingent on the resistance (rigidity) of the matrix and which leads to growth of the nascent adhesions into mature focal adhesions (FAs) [55,56]. The classical route of reinforcement involves talin stretching, which exposes binding sites for vinculin [57]. The latter, when engaged, provides an additional link to actin, thus reinforcing the integrin-actin connection. Another mechanosensitive step during these local contractions involves the recruitment of α-actinin, which also strengthens the integrin-actin link and leads to further force transmission [55,58,59]. Therefore, if the matrix is too soft, there is insufficient force accumulation in the time required for adhesion reinforcement to occur, thereby leaving the adhesions small, or leading to their disassembly. In parallel to the local contractions, there is a continuous flow of F-actin from the cell edge inwards; the centripetally moving actin fibers engage with the adhesions, and the extent of this engagement can control the magnitude of force transmission to the matrix [60,61,62]. Thus, if the matrix is stiff enough to provide resistance to the early actomyosin contractions and/or the flow at early times, reinforcement will occur through the talin-vinculin axis, and larger forces will be transmitted from the continuously flowing actin to the matrix. When the adhesions grow further, they are typically present in lamellar regions, 2–3 µm inwards from the cell edge, and force transmission becomes actin flow-independent [63]. This is likely because at this stage the adhesions are connected to more complex actin structures, such as actin stress fibers or their precursors; indeed, this independence on flow is favored on stiffer matrices [63]. Notably, the temporal loading of force through such structures does not depend on matrix rigidity, but rather on the intrinsic contractile activity that the cell generates [39,64]. This indicates that the long-term forces transmitted from the cytoskeleton to the matrix through individual adhesions are surprisingly non-mechanosensitive. The degree of force transfer does, however, depend on the local density of F-actin near the adhesion sites, and likely also on the organization and complexity of the actin networks [64].

**Figure 1:**
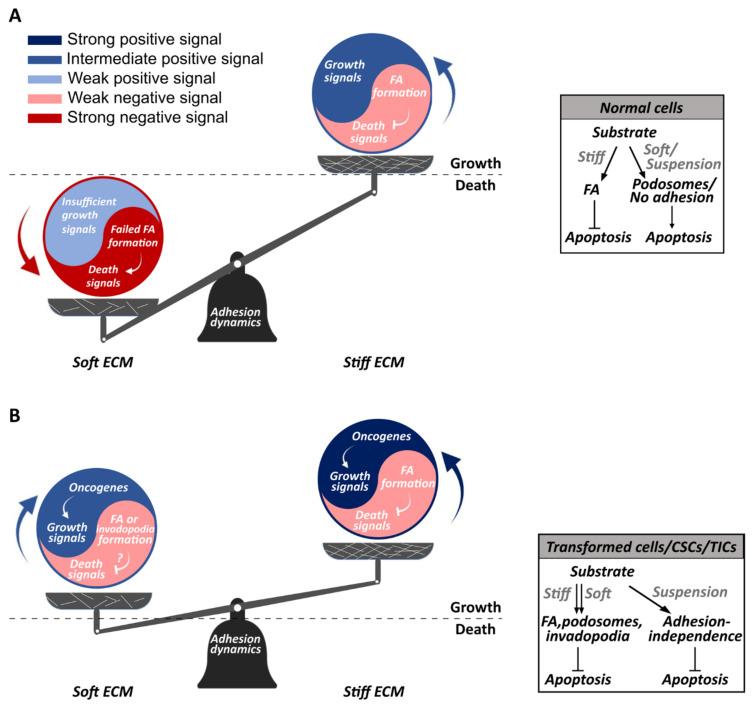
The ‘yin and yang’ of cancer cell growth and mechanosensing. An illustration of the relationship between ECM rigidity and cell growth, where ‘yin’ (red) represents the cellular response to negative mechanical stimuli (e.g., a soft ECM), and ‘yang’ (blue) represents positive growth signals (see figure key). (**A**) In normal cells, the negative mechanical signals from a soft ECM are stronger than the positive signals, causing the cells to undergo anoikis. (**B**) In anchorage-independent transformed cells, the balance shifts and cells grow regardless of ECM rigidity.

Altogether, in normal (non-transformed) cells, the stepwise, highly regulated process of mechanosensing leads to distinct phenotypic differences between cells that are exposed to soft compared to stiff environments. This occurs due to the tight connection between the adhesions and the cytoskeleton, and includes effects on various cellular features, including cell size, polarity, stiffness and the cytoskeletal connection with the nucleus [65,66,67]. These are accompanied by the activation of signaling cascades that affect the most fundamental cellular functions. Specifically, non-transformed cells activate proliferation pathways on stiff matrices but inhibit them on soft substrates. However, as mentioned above, cancer cells ignore matrix rigidity and can grow on stiff and soft matrices (Figure 1). This highlights the transmission of mechanical signals into biochemical cues within the adhesions, typically referred to as ‘mechanotransduction’, which might be altered in cancer cells.

## 5. Mechanotransduction at Focal Adhesions

The common view of the mechanism by which high rigidity induces normal cell growth includes two major aspects: 1. The formation of large adhesions, which transmit high forces, leads to abundant activation of mechanosensors and high activation of growth signals [50,68]; 2. The assembly of ‘actin cap fibers’ (thick actin stress fibers that connect between these adhesions and the nucleus) assists in translocation of growth-promoting transcriptional regulators into the nucleus, in particular YAP/TAZ [67,69]. Both processes are closely linked to the spreading of cells to large areas, which is a typical characteristic of non-transformed cells on stiff matrices. Importantly, however, cancer cells appear to not conform to these rules. Anchorage-independent cancer cells often produce small adhesions and have small areas [55,70], and they rarely produce actin cap fibers [71]. Still, cancer cells are generally highly proliferative on both stiff and soft surfaces, and they are able to produce high forces on both. This suggests that the determination to enter a proliferative state cannot be explained solely by the cell areas, adhesion sizes or presence of actin cap fibers. Rather, the modes of signal accumulation via adhesions, regardless of their sizes, are of great importance to the initiation and preservation of a proliferative state. Here, we discuss two major signaling hubs—the FAK/Src complex, and Rho family GTPases—and the ways by which they might contribute to cancer cell growth even through small adhesions.

### 5.1. The FAK-Src Complex

The recruitment and activation of FAK in integrin adhesions is one of the early stages in the cellular response to external mechanical stimuli. Activation of FAK at the membrane is initiated by the autoinhibited FERM-kinase complex (inactive FAK) binding to PIP2-enriched plasma membrane regions, and undergoing PIP2-induced conformational changes [72]. PIP2 can induce FAK clustering and enhance its autophosphorylation on the Y397 site by preventing FAK from forming a fully closed conformation. The phosphorylation of tyrosine 397 in FAK increases the accessibility of Src to residues Y566 and Y577, thus enhancing Src-mediated FAK activation [73], and forming a dual kinase complex. The activated FAK-Src complex can then bind to and phosphorylate its two major downstream effectors—p130Cas and paxillin, which primarily act as scaffolding proteins. The resulting integrin adhesion signaling center can activate or recruit downstream effectors such as Crk, CrkL, RASGap, DOCK180/ELMO, which then activate Rac/CDC42/RhoA, thereby triggering diverse signaling pathways responsible for FA turnover, actin cytoskeleton remolding, cell spreading and cell migration [74,75]. FAK/Src are also mediators of multiple cell growth and survival signaling pathways such as PI3K/Akt, Erk/MAPK, JNK pathway and NF-kB pathway, which demonstrates both their versatility and their role in transducing signals from the ECM and cell surface to cytoplasmic and nuclear events [48,76,77,78]. Importantly, as regulators of adhesion dynamics, and as very early constituents of nascent adhesions, FAK and Src could confer these effects even through small adhesions. Thus, in cases of continuous assembly/disassembly cycles that are observed in cancer cells, signals can still be accumulated over time via activation of this complex.

Moreover, as demonstrated by many studies, FAK and/or Src are highly expressed and/or activated in many cancers [79,80,81,82,83,84,85,86,87,88]. Herein, we have summarized the involvement of FAK and Src, as well as other major growth-associated signaling pathways, in the top 5 deadliest cancers (Table 1). Hyperactivation of FAK/Src signaling can help cancer cells to promote cell survival either in the absence or presence of “negative” signals from ECM-integrin adhesions (e.g., a soft matrix). In addition to promoting cancer cell proliferation, FAK and Src play vital roles in cell invasion and metastasis, making the cancer more aggressive and conferring poor patient prognosis. Indeed, overactivation of Src and/or FAK perturbs the integrity of FAs and cell-cell adhesions and triggers invadopodia formation, which is crucial for metastasis in a variety of cancer cell lines [89,90,91]. Therefore, FAK and Src are potentially promising therapeutic targets, as evident from the numerous clinical trials that have been carried out over the years [78,92].

### 5.2. Rho Family GTPases

Actin polymerization is required for rigidity sensing, and this involves Rho activation. In 1992, Ridley and Hall showed for the first time that Rho is essential for regulating the assembly of integrin adhesions and actin stress fibers induced by growth factors [120]. Their work drew attention to Rho GTPases, and to date 20 Rho family members have been identified. They can be divided into 5 groups, based on primary sequences and known functions: the Rho-like, Rac-like, Cdc42-like, Rnd, and RhoBTB subfamilies. Rho family proteins integrate broad upstream regulatory inputs to produce broad effector outputs and directly or indirectly contribute to almost all cellular activities, the most significant of which being actin polymerization and stress fiber formation. For example, ROCK (Rho-Kinase, a downstream effector of Rho) can enhance myosin activation by increasing myosin light chain (MLC) phosphorylation, which promotes myosin contractility. This generates tension and drives the feed-forward loop that promotes the formation of stress fibers, as well as the maturation and growth of the adhesions (in non-transformed cells). Another Rho effector, the mammalian homolog of diaphanous (mDia), is also required for the modulation of this process [121]. Rac and Cdc42 activate the Arp2/3 complex through WAVE and N-WASP proteins, respectively, thus promoting actin polymerization [122].

Importantly, Rho GTPases are major players in cancer progression, and are specifically involved in processes of cell transformation, tumor growth, angiogenesis, invasion, metastasis, and resistance to cancer therapy. Cancer cells are characterized by perturbed cytoskeletal architecture, along with dysregulation of Rho GTPases. The altered expression or activation of several Rho GTPases has been reported in a variety of human tumors. As Rho GTPases have many effector molecules and are involved in numerous signaling pathways, it is conceivable that some of these effectors and pathways are oncogenic, whereas others have tumor-suppressive effects [123]. The roles of Rho GTPases as either oncogenic or tumor-suppressive in cancer progression are cell line-specific and context-dependent. For example, shRNA-mediated silencing of RhoA and RhoC inhibited the proliferation and invasiveness of MDA-MB-231 triple-negative breast cancer (TNBC) cells in vitro and in vivo [124]. In the same cell line, stress fiber assembly and FA formation was decreased in clones stably expressing RhoA siRNA and RhoC siRNA. These clones also displayed reduced invasion, motility and growth rate [125]. However, the opposite was observed in another study, as stable RhoA knockdown in TNBC cells led to the development of significantly more lung metastases in mice, compared to cells treated with control shRNA and dominant negative RhoAT19N allele [126]. Similar observations were made in TNBC cells containing a deletion of ARHGAP18, a RhoGAP family member. This deletion resulted in increased RhoA activation, enhanced actin stress fiber and FA formation, and reduced cell proliferation, migration, tumor growth and metastasis [127]. A plausible explanation for the contradicting roles of RhoA in cancer progression is that RhoA may have preferred effects on certain downstream effectors, which are highly context-dependent (the contradicting roles of Rho GTPases in breast cancer are reviewed in detail by Humphries, Wang and Yang 2020 [128]). Nevertheless, the central role that Rho GTPases play in adhesion dynamics endows them with the capacity to drive growth-promoting signals through either large or small adhesions.

## 6. Anchorage-Independence and Mechanosensing Aberrations Characterize Metastatic Cells

There are two main models which attempt to elucidate the heterogeneity, initiation, and metastatic potential of tumors. The first is the clonal evolution model [129], which proposes that somatic mutations in a normal cell transform it into a neoplastic cell. A further accumulation of mutations gives rise to new clones, and selective pressures favor and enrich the metastatic populations. The second model is the cancer stem cells (CSCs) hypothesis [130,131], which posits that tumors arise from a rare subset of CSCs that possess self-renewal abilities, resistance to drugs and radiotherapies, and can generate cells with greater metastatic potential than their non-stem cell counterparts. Cancer cells characterized by their ability to form tumors following transplantation in immunocompromised mice, as wells as driving tumor growth and metastasis are known as tumor-initiating cells (TICs) [132]. Notably, these two models can be seen to be complementary, rather than mutually exclusive [133]. Importantly, matrix stiffness has been shown to affect the proliferation and stemness of CSCs, as well as the enrichment of TICs in multiple cancer types [134,135,136,137,138]. Still, one of the central characteristics which enable TICs and/or CSCs to drive metastatic progression is the deregulation of anoikis, which manifests as anchorage-independence. This phenomenon was described as a characteristic of transformed cells as early as the 1950s [139,140], and naturally, it hinted to the existence of a mechanistic link between anchorage-independence and cancer metastasis, especially when considering the journey of metastasizing cells.

During metastasis, cancer cells undergo a series of stages, and failure to complete any one of them can terminate or delay the process [141]. First, the metastatic cells detach from the primary tumor and invade the surrounding tissue, which requires the promotion of cell motility, EMT, and secretion of microenvironment-modulating factors [142]. Next, they infiltrate and travel through the circulatory system as circulating tumor cells (CTCs), arrest in distant capillary beds, and finally extravasate into the parenchyma of a distant organ and colonize it [143,144]. All of this requires the cells to exhibit characteristics which allow them to evade normal regulatory mechanisms, as outlined in ‘The hallmarks of cancer’ [145,146]. Anchorage-independence in particular, allows metastasizing cells to survive in suspension as CTCs, under adhesion-deficient conditions, and to colonize organs with a stiffness dissimilar to their tissue of origin [147]. Studies confirm that signatures and phenotypes that characterize anchorage-independent growth also serve to identify metastatic tumors [148]. Metastatic cells may employ several strategies to overcome anoikis, such as: counteracting negative inputs, activating survival signals, undergoing EMT, triggering integrin switching, or entering dormancy [149].

Importantly, it has recently been shown that several of these strategies are associated with mechanotransduction aberrations [150,151,152]. In particular, anomalous mechanosensory elements cause cancer cells to apply improper forces and to detract from or alter the influence of the ECM rigidity on cell phenotypes. Thus, a link between aberrant mechanosensing and anchorage-independence has been established, strengthening the understanding that anchorage-independence is a mechanobiological phenomenon. In fact, in some cases, mechanobiological pathways were shown to be dominant over biochemical pathways in determining whether the cells are anchorage-dependent, including in cancer cells that express mutant forms of oncogenes [70].

A prime example of this is the role of tropomyosins in cancer progression. Tropomyosins (Tpms) are key regulators of actin structure dynamics, and thus have an influence on cellular structure and function, e.g., morphogenesis, proliferation, and biomechanics [153]. Tpms are coiled-coil parallel dimers that form head-to-tail homopolymers along actin filaments. There are over 40 identified Tpms in mammals, which regulate the interactions of actin filaments with myosin motors and actin-binding proteins in an isoform-specific manner [154]. Actin filament nucleators, such as formin and the Arp2/3 complex can affect actin organization and its interactions with actin-binding proteins, including specifying which Tpm isoform binds to the actin filament [155]. Additionally, the assembly of complex structures, such as stress fibers and podosomes, involves the collaboration of multiple types of actin filaments, characterized by their specific Tpm components [156]. Notably, Tpms are known to be sensitive markers of cellular transformation [157]. Several studies have shown that transformed cells lack Tpm 2.1 expression, and that restoring the levels of Tpm 2.1 or Tpm 1.6 can re-establish proper mechanosensing activity and revert the cells to an anchorage-dependent state [70,158,159]. In normal cells, Tpm 2.1 mediates cytoskeletal reorganization through Rho kinase, and induces anoikis through intrinsic apoptosis, in a caspase-dependent fashion. Interestingly, Tpm 2.1-transduced cancer cells undergo anoikis in serum-free as well as in normal growth conditions, suggesting that the growth factor-derived signals did not overcome its adhesion-dependent signaling [159,160]. Tpm 3.1 is another important isoform that plays a role in cell motility [161] and proliferation through the MAPK pathway [162], and is implicated in transformation, as tumor cells retain its expression [163].

## 7. Which Mechanobiological Processes Underlie Anchorage-Independent Cancer Cell Growth?

Altogether, the studies in recent years on the mechanobiological features of cancer showed that the ability to evade anoikis and to proliferate uncontrollably are key mechanobiological processes that are misregulated in cancer cells. Through such processes, intricate signaling networks are manipulated, leading to altered cancer cell fate. The system by which cells either grow or die due to mechanical stimuli mirrors a ‘yin and yang’ relationship. The ‘yin’ may be seen to represent negative growth signals from cell-ECM interactions (soft ECM), while the ‘yang’ symbolizes positive growth signals (Figure 1). In non-transformed cells that properly sense ECM rigidity, the ‘yin’ element is dominated by signals that support cell death on soft matrices. However, in cancer cells, ‘yin’ represents inhibition of negative growth signals from cell-ECM interactions, while ‘yang’ symbolizes amplification of positive growth signals from oncogenic signaling molecules, which results in cancer cell growth, regardless of rigidity (Figure 1).

Inhibition of negative signals can occur through numerous mechanisms, including alteration of the integrins themselves. For example, integrin signaling is not always limited to FAs, but rather can take place through endosomal signaling, wherein FAK is recruited to endosomes and is activated; this mechanism was shown to correlate with reduced anoikis sensitivity and anchorage-independent growth [164]. Another mechanism involves the role of death-associated protein kinase 1 (DAPK1), a central apoptotic regulator that interacts with tropomyosin and talin, and can be recruited to adhesions [165,166]. In normal mouse fibroblasts, when the mechanical feedback forces from a soft ECM are insufficient for the maturation of FAs, DAPK1 dissociates from the adhesion complex and triggers apoptosis [167]. However, in transformed cells, DAPK1 activity is inhibited, thus preventing the onset of apoptosis in the same non-adhesive conditions [168].

Hence, it appears that proper responses to negative signals in non-transformed cells (i.e., activation of death on soft matrices) are inherently linked to the state of the adhesions, and that ignoring such signals by cancer cells involves alterations in the mechanobiological processes that affect or are affected by the adhesions. Particularly, a switch between adhesion types may be at the heart these alterations (Figure 1). Normally, the inability of integrin adhesions to mature on soft surfaces can lead to the formation of podosomes rather than FAs [169]. Podosomes bear high similarity to invadopodia, which are found in many transformed cells and which require high activation of FAK, Src and PI3K [169,170]. This hints at a connection between the two major cell-ECM adhesion types—FAs and podosomes/invadopodia, and suggests that suppression of negative signals by cancer cells could involve a shift to the latter type. Indeed, a recent study provided strong support for this connection by showing that FAs and podosomes/invadopodia are switchable, in a process mediated by KANK family proteins [171]. KANK connects microtubules to the integrin-ECM complex, and suppresses the release of GEF-HI (an activator of Rho GTPases) from microtubules. Low levels of Rho/ROCK activity results in few actomyosin contractions at the integrin-ECM complex, which is permissive for the formation of podosomes/invadopodia, but not FAs, as in the case of cells on soft surfaces. In contrast, when KANK dissociates from microtubules, the elevated Rho/ROCK activity leads to FA formation rather than podosomes/invadopodia [171]. Notably, KANK1 promotes apoptosis and is downregulated in many cancers [172]. Thus, evading anoikis on soft surfaces could occur through a mechanism that both inhibits apoptosis and involves changes in the adhesions due to absence of KANK proteins. This hypothesis remains to be tested.

The ‘yang’ component involves crosstalk between many signaling pathways, which form an elaborate and intricate network. The previously mentioned oncogenic signaling molecules, Rho family GTPases, FAK and Src, are central mediators in this signaling network. For instance, in Ras-transformed cells, hyperactivated RhoA suppresses p21 and promotes cell growth through the MAPK pathway. Interestingly, the sustained MAPK signaling resulting from the Ras oncogene decreases the activity of ROCK, which is downstream of RhoA. This does not induce actin stress fiber formation, and it increases cell motility [173]. In addition to the signals from integrin-ECM adhesions, FAK/Src also respond to growth factors and their receptor tyrosine kinases (RTKs), which are both dysregulated in many cancers. For example, elevated expression levels of epidermal growth factor receptor (EGFR) are related to tumor malignancy and drug resistance [174]. Src and activated EGFR form a complex that synergistically promotes DNA synthesis, cell growth in soft agar and tumor formation in nude mice [175]. The co-localization of FAK and ErbB2/3 at cell protrusions is essential for ErbB-induced Src-MAPK signaling activation and cell transformation [176]. Moreover, EGFR inhibition abolishes anoikis resistance in intestinal epithelial cancer cells, due to the disruption of FAK-Src interactions and the downregulated activation of MEK/MAPK and PI3K/Akt signaling [177].

Despite many years of study, and the clear conceptual link, understanding of the involvement of adhesion and mechanobiological processes in tumor growth is still poor. Here, we summarize the involvement of adhesion types, signaling pathways, and rigidity dependency in lung, breast, and pancreatic cancer in order to highlight the complexity and diversity of these processes (Table 2). We propose the ‘yin and yang’ relationship as a conceptual framework that could help in studying these relations, with particular focus on the shift in adhesion types in cancer cells.

## 8. Conclusions

There exists a paradox between “grow” and “invade” in cancer cells, as highly metastatic cells display expression levels of proliferation-related genes that are inversely correlated with the expression of invasion-related genes [191]. However, interestingly, proliferation/survival and invasion all require the elevated activity of the Rho family GTPases, FAK and Src signaling hubs. It is possible that once cancer cells succeed in surviving and growing on a soft ECM by manipulating the ‘yin and yang’ equilibrium, the high levels of Rho family GTPases, FAK and Src cause them to shift towards a more invasive and metastatic state by altering the adhesions to invadopodia.

It is well established that anchorage-independence characterizes metastatic cancer cells, yet it is equally true that matrix stiffness affects the behavior of anchorage-independent cells, e.g., by modulating proliferation, migration, dormancy, and metastatic progression. Their varying responses to different rigidities suggest that cancer cells do not completely disregard the rigidity, but rather experience a functional shift, which alters their sensitivity threshold and ultimately, their responses to physical stimuli. Thus, anchorage-independence does not signify anchorage-insensitivity. This seemingly paradoxical paradigm emphasizes the need to refine our understanding of the balance between ‘anchorage-independence’ and ‘stiffness preference’. Further investigations centered around the relevant cellular mechanisms will help to elucidate this central question of mechanobiology.

## Figures and Tables

**Table 1:** Major growth-associated FAK and/or Src signaling pathways in the top 5 deadliest cancers according to the American Cancer Society.

Cancer Type	Species	Elevated Kinase Activity		Growth-Associated Signaling Pathways	References (PMID)
		FAK	Src		
Lung	Human	+	+	PI3K/Akt	[93,94]
	Human		+	MEK/MAPK	[94,95]
	Human		+	YAP/Hippo	[96,97,98,99]
Colorectal	Human	+	+	PI3K/Akt	[100]
	Human		+	EGFR	[101,102]
	Human, rat		+	EGFR/ERK	[103]
	Human		+	ERK	[104]
Breast	Human		+	STAT3	[105]
	Human	+	+	HER receptors/PI3k/Akt /MAPK	[106,107]
	Human		+	YAP/Hippo	[108,109]
Pancreatic	Human	+	+	Ras/Raf, PI3K/Akt	[81,83,110]
	Human, mouse	+	+	ERK	[108]
	Human		+	EGFR/Erb2, ERK, STATs, TGF-β	[111,112,113]
	Human, mouse		+	EGFR/STAT3	[114]
Prostate	Human, mouse		+	SFK (Src family kinases) Lyn	[80]
	Human	+		PI3K/Akt	[115]
	Human		+	EGFR/Akt/ERK	[116,117,118]
	Mouse		+	MAPK	[119]

**Table 2:** Adhesion structures and related signaling pathways in different cancer types.

Cancer Type	Adhesion Types	Adhesion Rigidity Dependency	Signaling Pathways	Cancer-Related Effects	In Vitro/In Vivo/Ex Vivo	Publicat-ion
Lung	FA	N/A	VAV2/FAK/Rac1	Promotion of metastasis	In vitro: human H1299 and H460 cells In vivo: transplantation in nude mice	[178]
	FA	Promotion of FA formation on soft ECM	FAK	Increase in migration velocity and distance	In vitro: human A549 cells	[179]
	FA	N/A	Keap1 upregulation of RhoA activity	Inhibition of cell motility caused by FA turnover inhibition	In vitro: human A549 cells	[180]
	FA	Increase in FA formation and size on stiff ECM	N/A	Decrease in cell motility	In vitro: human H1299 cells	[181]
	FA & invadopodia	N/A	StarD13/RhoA/ Rac1/FA, SrarD13/Cdc42/ invadopodia	Inhibition of cell motility (immature FA); promotion of cell invasion (invadopodia)	In vitro: human A549 cells	[182]
	Invadopodia	N/A	Cortactin/ Cdc42/N-WASP	Promotion of cell invasion	In vitro: human H1299 and A549 cells	[183]
Breast	FA	Increase in FA formation on stiff ECM	Integrins/PI3K/Akt	Promotion of cell invasion and malignancy	In vitro: human MCF10 and Ha-ras MCF10 AT MEC cells In vivo: MMTV-Neu mice model, transplantation in mice	[29]
	FA	Increase in FA assembly and size on stiff ECM	ERK/Rho/ Src/FAK	Increase in cell growth and perturbation of tissue architecture	In vitro: Human HMT-3522 S1 cells In vivo: Transplantation in transgenic mice	[184]
	FA	No difference in FA areas across rigidities	N/A	Increase in cell proliferation	In vitro: human MDA-MB-231 cells	[70]
	Invadopodia	Increase in invadopodia quantity and activity on stiff ECM	Rho/p130Cas/ FAK	Promotion of cellular invasion	In vitro: human MCF10CA1d cells	[185]
	Invadopodia	Increase in invadopodia formation at ~30 kPa and 1.8 Gpa	N/A	Increase in ECM degradation	In vitro: human MCF10CA1d cells	[186]
	Invadopodia	Decrease in invadopodia formation in stiff 3D networks	Rac1/ROCK	Increase in cell migration	In vitro: human MDA-MB-231 cells	[187]
Pancreatic	FA	No difference in FA areas across rigidities	N/A	Increase in cell proliferation	In vitro: human PANC-1 cells	[70]
	FA	N/A	Inhibition of FA turnover by cAMP	Inhibition of cell migration	In vitro: human PANC-1, BxPC3, Capan-2, MiaPaca-2 and SUIT-2 cells	[188]
	FA & invadopodia	N/A	Src/FAK/ p130Cas (FA); Src (invadopodia)	Promotion of cellular invasion and ECM degradation by FA	In vitro: human PANC-1 and BxPC3 cells	[189]
	Invadopodia	N/A	N/A	Presence of invadopodia within human tumors	In vitro: human PANC-1, BxPC3, Capan-2, MiaPaca-2, SU86.86, MRC-5 and L3.6pl cells Ex vivo: human tumor surgical specimen	[190]

FA, focal adhesions; N/A, not available—data not found in study.
